# There Are Conscious and Unconscious Agendas in the Brain and Both Are Important—Our Will Can Be Conscious as Well as Unconscious

**DOI:** 10.3390/brainsci2030405

**Published:** 2012-09-18

**Authors:** Lüder Deecke

**Affiliations:** Department of Clinical Neurology, Medical University Vienna, Vienna 1090, Austria; Email: lueder@deecke.com; Tel.: +43-1320-5558; Fax: +43-1320-0309

**Keywords:** consciousness, the unconscious, free will, conscious free will, free decisions

## Abstract

I have been asked to write a few words on consciousness in this editorial issue. My thoughts on consciousness will focus on the relation between consciousness and will. Consciousness is not an epiphenomenon as some people believe—it is not a psychological construct either. Consciousness is a brain function. With deeper thought it is even more than that—a brain *state*. Writing this, I am in a conscious state, I hope at least. In every day philosophy, a close connection of consciousness with will is ventured, and is expressed in the term “conscious free will”. However, this does not mean that our will is totally determined and not free, be it conscious or unconscious. Total determinists postulate total freedom from nature in order to speak of free will. Absolute freedom from nature is an *a priori* impossibility; there is no way to escape from nature. However, we have relative freedom, graded freedom, freedom in degrees, enabling us to make responsible decisions and be captains of our own destiny. We are not totally determined. We can upregulate our degrees of freedom by self-management or we can downregulate them by self-mismanagement. In the present communication consciousness and the unconscious are discussed in their various aspects and interactions.

## 1. What Is Consciousness?

Consciousness is a complex, comprehensive brain function and even a brain state. Some believe consciousness is only in humans and not in animals. I believe there is also consciousness in higher animals. I am convinced consciousness is a pre-human achievement of the evolution; we can ask the question, why has consciousness come into the world. If we think about this, we find at least two reasons why in evolution nature had to “invent” consciousness. To have consciousness was a matter of survival. In evolution brains became more and more complex, and to survive brain processing had to be fast. So in the sense of Darwin [1,2] the fittest was a being that could act and react quickly. This can only be achieved by means of data reduction. Between the information flow in our senses and that in our consciousness, there is a selection—and this sophisticated selection (intelligent filtering, concentration on the important matters, supported by our immense memory) is also unconsciously organized—an enormous compression of information takes place, a data compression of several orders of magnitude (at least 10^4^). In information theory the term “channel capacity” can be defined, and it can be measured in bit/s. Through the receptors and afferent nerves at least 10^5^ bit/s (order of magnitude) are flowing, in consciousness, however, only 10 bit/s show up [3]. This is the information compression, that has helped us to survive and thanks to it we remained capable of acting. We gain a lot of safety out of this because we can trust that our unconscious selection routines are intelligent and work reliably so that nothing is withheld from consciousness what we actually ought to know. Consciousness is like a platform or stage or screen in the brain; it is like the executive floor of a company, where only agendas ready to be signed are let up, but the groundwork has already been done by the *unconscious* pre-processing. So consciousness is the highest “authority” so to say. And talking of leadership on this top level, it is the will, the human will that here is the ruler. The will always takes part, albeit in that it delegates as much as possible to unconscious routines and expert systems.

But there are two evolutionary principles that have shaped consciousness, the first being the need of data reduction and what is the second principle? It is the hierarchical organization of the nervous system. Nature is “constructing” with a strategy different from that of an architect (the old house is destroyed and a new house is built on the lot) nature does not destroy the old system but creates a new one with inhibitory properties on the old system. So even we as recent human beings have the old systems in us, a fish brain, a reptilian brain and a mammalian brain. The most important feature of hominid evolution is corticalization, and what is more evolution of the association cortex. The association cortex is twofold, 50% of it is retrorolandic and 50% is prerolandic. The prerolandic association cortex is the frontal cortex. Kornhuber and I have shown that it is the frontal cortex, in particular the pre-frontal cortex which can be regarded as the functional topography of the will [3,4].

Part of this pre-conscious filtering or selection/data reduction is taking place already on the spinal level, but most of it in the forebrain. Attention is important and works on its own. But selective attention is definitely steered by the will. Also selective attention needs the “hub”, where the converging sensory messages meet with motivational impulses from the limbic system and the frontal brain, in order to select and make the right choice to separate the wheat from the chaff and to allow only the important information to be stored in memory [5,6]. Even such elementary processes like saving into the memory stores are highly “limbicly loaded”: Without motivation and emotion not much is registered, and even less can be recalled. And what is more emotional engagement helps in learning! We cannot cram examination knowledge into us during sleep. We need consciousness for this with focused attention and concentration governed by our will. Week-willed people are usually highly distractible. Whether consciousness is necessary for learning or not, depends on the kind of learning we have in mind. With procedural learning (motor learning, learning of behavior) consciousness may not be an absolute prerequisite (although also here it helps if I consciously concentrate on the task). For the new acquisition of skills we need the alert attention and concentration while learning in any case. What the declarative learning is concerned, we think it needs the focused concentration to learn. Otherwise I get into trouble with distraction, and I know from my daily life when my thoughts go astray, I am not capable of doing my home work properly. And what is more: What is learned under these suboptimal conditions is quickly forgotten. 

We see, consciousness under these circumstances is not at all powerless but essentially important and indispensable, because it enables activity which is inspired by knowledge and problem solving with an overall view. A brain-injured patient, who does not regain consciousness, cannot just by living for a long time in this state compensate for his deficiencies, but he can, if he regains consciousness. The brain is an immensely complex composition, in which many things are tried out spontaneously. What is going to be durable depends upon what passes, in the light of consciousness, the test in the outside world. Without this permanent corroboration, the brain would be ungovernable. The realization that consciousness is so important, is not only due to the fact that it facilitates learning, selection and freedom but also due to the fact that it does not do everything alone but it delegates most agendas to unconscious subroutines that have been available in the realm of living beings long before man’s existence and which lead, if necessary, again to cooperation with consciousness. Burdening consciousness with the entire information processing would mean to overcharge it completely. The fact that it is, among the causes in the brain, not under the first ones temporally, does not lessen its efficiency—on the contrary, it means that it remains undisturbed by many random swayings of neuronal activity and is protected against quite a few false alarms by many pre-placed thresholds and filters, so that it can devote itself to the really important projects. The creativeness of man, however, to which consciousness contributes so much, has made man a danger to the life on earth; he has already caused the extinction of many species of living beings. Thus, the devaluation of consciousness (for instance declaring it an “epiphenomenon”) is not an appropriate way of dealing with it. What is necessary is a humane education and making use of it in a wise manner (regarding consciousness as an “epiphenomenon” *cf.* [7,8,9]). 

What is going on in our brain when we are going to act or make a decision? I mean the foreperiod during which the readiness potential occurs: Standing immediately prior to a decision, already driven by will, but reflecting and perhaps inner struggles and then insight; after the planning and the decision there is—despite the delegation of many details to subprograms which were overlearned and then became unconscious again—purposeful vigilance, care, thoroughness, corrections, will of completion and new plans: all this belongs to will. The crucial final hurdle is the decision. Power of decision above all belongs to will, but stamina is also important. Prior to all this there is already openness to the world, active searching, perceiving, considering and thinking, the manifold mental interests which already begin in infants when collecting leaves or shells.

Experimental Psychology professionally began with Wilhelm Wundt [10] who founded the first psychological laboratory and who was primarily psychologist of will and who distinguished will as the selector between the drives. In this experimental tradition it is predominantly Narziss Ach, who has to be named [11,12]; he combined the method of systematic introspection with the measurement of reaction times. Through this, and other tests, he came upon the high effort of will for inhibition, which was necessary to overcome previously learned responses. It was also Ach, who pointed to unconscious components of will as a consequence of cerebral automation. “In no field of psychology is there greater confusion than in the field of will”, Ach wrote in 1910 [13]. Kurt Lewin [14] and Rohracher [15] introduced further methods; Rohracher, for instance, investigated persons, who suppressed their hunger by will-power.

The deeper cause of the confusion Ach wrote about was the discovery by Wallace and Darwin, that the mind of man is the result of evolution. Man was now grouped with monkeys and apes as a primate. Behaviorism and Freudism in the USA drew rather one-sided conclusions from that assumption for the problem of the will. John B. Watson, in his behavioristic manifest of 1913 [16], threw overboard not only will but consciousness as well as subjects of Psychology. His program was restricted to terms like stimulus, reaction and adaptation. In this field, prepared by behaviorism, Freudism had an easy job. Freud negated will and considered the ego a weak link between the drives and the over-ego or super-ego; he excluded freedom. The belief in the Freudian dogmas (e.g., the “anal character”, which, after 1968, advanced to a compulsory examination requirement for medical students) and the use of a brutalized language (e.g., “*Objektbeziehungen*”, object relations) has decreased by now. 

Some passages of the present manuscript—the ones with relation to consciousness—were taken from the book in print: Hans Helmut Kornhuber (1928–2009) and Lüder Deecke “*The Will and Its Brain—An Appraisal of Reasoned Free Will*” [3]. 

## 2. The Unconscious

Sigmund Freud is still regarded as the “discoverer of the unconscious” (and of such processes as repression and sublimation) which is wrong. Ebbinghaus already said: “What is new with these theories is not true, and what is true is not new.” HJ Eysenck ([17], p. 36) cites this statement of Hermann Ebbinghaus (1850–1909). The unconscious was already known to the ancient world, also to Thomas Aquinas, more so to Leibniz and initially in the Romantic period, to Carus [18]. 

Based on both—Carus and Schopenhauer [18,19,20]—Eduard von Hartmann wrote his multi-volume work “*The Philosophy of the Unconscious*”, which appeared in 1868 [21] and had great influence. Anyway, Freud never claimed to have discovered the unconscious [22], and as far as the psychodynamics are concerned, Freud, without admitting it, took the essential insights from Nietzsche; compare Freud and the list of psychological discoveries of Nietzsche, given by Jaspers [23]. We are now in a position to say a little about Freud’s conception of the unconscious. This is Freud’s term: *das Unbewusste*, the unconscious. The term *Unterbewusstsein*, the subconscious was later introduced by others. The reason may be that the word “unconscious” in the English language has strong meanings that Freud did not have in mind, namely powerless, passed out, fainted, having a black out, *etc*. The term subconscious does not elicit such associations. Be that as it may, Freud’s conception of the unconscious is odd from the beginning: The unconscious as a something that constantly plays tricks on me, makes me produce Freudian slips, *etc*. 

In reality there are conscious and unconscious agendas in the brain and both are important. It does not help to play the unconscious off against the conscious. In the brain at any time most of the agendas are unconscious, much of it is, however, consciousness-prone. The conscious and the unconscious always work together, not only in dreams but also in wakefulness. Our conscience for instance can admonish us out of the unconscious core from our memory, but by doing so it becomes conscious, and it is then the task of the reasoned will to draw the consequences out of it. In the system of drives, there are positive drives (such as care for children) and those that are counterproductive (such as envy and greed); the will must come along with them all and thereby makes the person community-fit and culture-fit.

Benjamin Libet concluded—on the ground of his somewhat bizarre “hybrid” experiments employing both the Bereitschaftspotential and introspection—that for the *control* of movements we have free will but for the *initiation* of movements or actions we do not have free will [24].

The argument for the total lack of freedom reads: The Bereitschaftspotential would prove the absence of consciousness with voluntary actions. However, the Bereitschaftspotential, being very small compared to the spontaneous brain waves, is the result of averaging over many stereotyped simple movements. The formation of will has already taken place prior to the start of the whole experiment, and the preparation for movement is initially handed over to unconscious routine processes of the basal ganglia, the supplementary motor area and anterior to it the pre-SMA [4], which in turn do the groundwork for the motor cortex, which finally gives the command for the single finger movement. However, even more remarkable is—not only in Libet’s experiments but generally—that consciousness is switched on about 200 ms prior to the onset of movement, for each of the many simple stereotyped movements of the index finger. At 200 ms before movement onset there is still time to make changes, if necessary [25]. This is a great expenditure for the brain and shows that even such unimportant movements are controlled, if they are voluntary. Consciousness with its brightness and its freedom is known to be restricted and its time is valuable. Only important events get access to it. Thus, 200 ms before movement onset consciousness is switched on so to say. This makes sense, and the reason for this is twofold: (1) to allow for “last minute changes” of the movement, small adaptive changes but also changes to the extent that I abstain completely from performing any act, *i.e*., to the extent of non action, Libet calls it veto; (2) The second reason why consciousness is switched on “last minute” is to enable learning, in order to improve performance. Also *cf.* Mele, A. [26] and Dennett, D. [9] for criticism of Libet.

## 3. Is Freedom Only in the Conscious World?

No, freedom is inherent in our brain. In the inner leadership by the will, in conjunction with the creative abilities of the frontal brain, there lies freedom. Freedom is necessary to find new solutions, to be creative and innovative. We mean freedom not against but with nature. An escape from nature is not possible. Also for mental activity, which is information processing by changing the order system, energy is necessary. Will does not stand above the brain, the mind is not positioned behind the brain. For example, in the case of an injury to the retina of the eye the visual brain (the visual cortex in the occipital lobe) sees the defect as a dark scotoma. In contrast, an acute loss of function of the whole visual cortex (by bilateral infarction of the calcarine artery) is not perceived: the patient is blind, without experiencing that he is blind (cortical blindness, also called “blindness of the soul”); he even denies his blindness and invents something should he be asked about the weather. Only days or weeks later does he learn that something is not in order with his eyesight, but he is unable to perceive this, in contrast to a hole in the retina. In the same way our emotional sphere does not live outside the brain, it is rather the inner aspect of the information processing in the human brain; conscious vision in man is a function of the cerebral cortex (in frogs it is still a function of the optic tectum of the midbrain, but frogs do not possess a consciousness that is similar to man). With our conscience it is similar, if this disappears secondary to a lesion of the frontal brain, the patient does not take notice that it has vanished; he can happily tumble into joke cracking (“*Witzelsucht*”) and other symptoms—by painful experience and practicing he can, however, gain insight. We bear in mind that in case of cortical blindness, the patient is not conscious of his blindness.

The school of Heckhausen and Kuhl confirmed that will is a complex function, beginning with consideration, planning and thereafter, decision, all this taking place in the bright light of consciousness and with self-critical connection to reality, then shifting parts of the processing into unconscious routines but with accompanying conscious control and, if necessary, corrections until the goal is reached. After Kornhuber [27,28] had already pointed towards different components and states of the process of will, psychological research of will led to a structuring of will into numerous states, which, if we see it correctly, didn’t prove very fruitful for education, therapy or the forensic assessment of responsibility. However, it could be shown neuropsychologically by examining patients with brain injuries that the selection of goals and the initiation of actions are distinguishable brain functions [29]. There were also animal experiments with single neuron recordings in the brain of monkeys [30,31] and new ideas came from cybernetics and artificial intelligence [32,33]. An international consensus conference of social researchers, psychiatrists, psychologists, lawyers and neurologists pointed at the urgency of research on will and volition with respect to the prevention of violence [34].

## 4. The Conscious and the Unconscious and Memory

The conscious/unconscious discussion has to be conducted re *memory* as well. Introspection shows us that our unconscious can address our memory and—this is the important thing—instantaneously it becomes conscious. An example: Our conscience (the moral instance in us) can admonish us out of the unconscious core from our memory, but by doing so it becomes conscious, and it is then the task of the reasoned will to draw the consequences out of it. Man does not have just one memory store, his memory systems are distributed. He also has different kinds of memory, episodic memory, working memory, *etc*. There is a working memory in the frontal lobe and in the basal ganglia (motor memory) and there is a working memory in the temporal lobe as well, with which we remember, e.g., telephone numbers for a short time or remember words while talking, until we have fitted them into the sentence. Structures of the limbic system in the depth of the temporal lobe are also important for the decision, which parts of all the information which flow through our consciousness shall be stored in the *long term memory*, for it would be counterproductive to remember everything. A brain has to be capable of acting quickly, and the larger a memory, the slower and more unreliable the recall. Only the most important information is, therefore stored for longer duration—an approximate estimation revealed that it is about 1% of the information flowing through our consciousness [35]. Before the evolution of the frontal brain, the limbic system decided what was important, and for this reason the basics of the fixation in the long term memory remained in the hippocampus. But surely also the *will* and the frontal brain have influence on the *selection for storage* and on the *recall* from long term memory [36,37], for not only the emotionally exciting events can be committed to memory, but we can also engrave our own goals in our mind over long periods, and our will guides our interests, our attention and our thinking, for which we need memory. The prefrontal cortex already influences the formation of the long term memory [36], but even more it takes influence on the retrieval of the memories we need for thinking, for self-criticism, and for the development of the personality [38,39,40]. The prefrontal cortex has strategic influence on the memory, on retrieval, storage, precision, care, in case of distraction by similar words, reliability of memorization regarding the sources, *etc*. [41]. Predominantly by bilateral frontal lesions with apathy, the formation of the long term memory is disturbed.

Similar considerations apply to the *dream sleep*, in which the brain is activated by random hits of impulses (stemming from a small assembly of nerve cells in the brain stem (Locus coeruleus), which has nothing to do with drives) and thus—being cut off from the world—produces bizarre stories: Self-development and self-maintenance of nerve cells by exercising in the dream sleep phase is a more realistic theory than that proposed by Freud, Jouvet [42] or Francis Crick (as in [28]), especially considering that the life of infants consists predominantly of dreaming. The activity of nerve cells goes hand in hand with the secretion of nerve growth factors (e.g., the brain-derived neurotrophic factor), the glia cells at rest do not only provide energy-supplying molecules, they also, in effect, clean the brain. As far as creativeness is concerned, however, one must not expect too much from dreaming. Great discoveries are made during the conscious mental work phase. Kekulé’s benzene ring was an exception. He did not have the idea during night sleep but during a short day dream only after intensely thinking about the problem.

## 5. Are the Amygdala Really That Important?

We would not consciously experience feelings, which primarily come from the limbic system, without the cortex, and here we mean the neocortex. The human achievement is the frontal cortex. This is the organ of control for many subcortical trends and flows. Mastering of them may result in happiness. However, not only happiness is initiated by the frontal lobe but also the sense of duty, taking care and many other feelings. These abilities can vanish after orbitofrontal lesions. The amygdala would signal us unconscious feelings of threat or danger by fellow species, it does the same in animals, where it is more important than in man: As known from patients with epilepsy surgery, even a bilateral excision of the amygdala causes symptoms only in the acute state, subsequently, however, compensation is rapid, so that the people affected are psychologically inconspicuous (see e.g., [43]). Only by means of special tests can it then be ascertained that they perceive threat in facial expression or in utterances of fellow men less well than control subjects [44]. This is in contrast of some scientists who nowadays hype the amygdala as the center of the soul and having the last word on all decisions, *etc*. Erroneously! If the amygdalae are excised in adult monkeys, they show less mistrust and are especially popular with their comrades [45]. This is different, when the development of social behavior is changed through bilateral excision of the amygdalae in monkeys in early childhood (2 weeks of age): the young animals obviously experience disappointment by too much carelessness and then become more anxious in company with their fellow monkeys; but by no means do they become autistic, they are otherwise inconspicuous in their social behavior [46]. Lesions that are restricted to the amygdala do not—in contrast to hippocampal lesions—cause memory disturbances [47]. We would not consciously experience feelings, which primarily come from the limbic system, without the cortex. The cingulate gyrus would at least be necessary.

Thus, the amygdala, a small assembly of neurons of about one and a half cubic cm in size [48], is a phylogenetically old organ (the archistriatum of the reptiles), which is less well protected against epileptic discharges than the neocortex. It is a frequent site of origin of temporal lobe epilepsy with semiconscious psychomotor attacks, which are often connected with brief, involuntary actions and linked predominantly with anxiety. In epilepsy surgery it is successfully excised, mostly together with the similarly old and seizure-prone hippocampus; however, the latter must be excised only unilaterally since bilateral excision causes memory loss. But in man, after unilateral interventions, one does not see changes of impulse control, no addiction, no sensation-seeking and no decrease in anxiety. For anatomical connections of the limbic system, see [49,50]. By the way, the amygdala is by no means in charge of all novelties; it specialized in the automatic detection of danger when dealing with fellow members of the species. For the general detection of novelties, which is of course essential to life, a much larger system is in charge of, it consists of the parietal cortex and the frontal lobe—including its limbic part (for the transfer of important information into long-term memory) which is the hippocampus [51]. Besides the automatic surveillance of dangers that come from fellow species members, there are also neurons in the amygdala that influence autonomous functions and control evaluations of smells [52]. 

## 6. If I Am Not Aware, Not Conscious of Something, I Have Agnosia, e.g., Anosognosia

Also this has a bearing on the conscious/unconscious debate. Anosognosia means that the patients have no conscious experience, that they are sick, that they have a disease. For instance a patient with Wernicke’s aphasia usually has anosognosia. Thus, this is a disorder of conscious experience of cognitive defects from brain lesions. It is interesting that anosognosia lasts longer with lesions of the parietal lobe [53], longest, however, after lesions to the prefrontal cortex, and what is more, in the orbital cortex as well as in the dorsolateral one [54]. This has also been confirmed for Alzheimer dementia [55]. Surely there is also a psychological tendency to obscure problems to oneself, but an organic dysfunction of will makes it difficult for the will for truth to come to insight and to take up the challenge, which is important for therapy and prevention [56]. The dilemma is clear: How can you treat a patient who has not the slightest conscious awareness of having a disorder or even a mere incapacity. The same applies for cortical blindness discussed in Section 3.

Gnosis and agnosis is of course all within the limits of natural sciences. Nevertheless a concept that considers *the mind* an independent being, independent from the brain, has been haunting the theories since Plato, and one believed him, the great and pure one, particularly since he linked it with immortality. Even Leibniz (“Monadology”), who was well versed in computers, thought the mind could not just be created by the cooperation of the parts of the brain. Plato’s theory even today—though in the meantime it has become much more abstract—calls the resistance of the realists, who then at times tend to throw—together with such “substance-mind”—also freedom overboard. Great influence he gained in modern age—not without some absurdity—by a scientifically thinking great brain, who thought that in the case of doubt he could trust solely on the consciousness of his own thinking. We are talking of Descartes [57]. His cogito ergo sum is part of his dualistic philosophy. The body, his concept says, is *spatial*; however, the mind is not. The *time-relatedness* of the mind is not entirely denied, for it must take effect on the body, but its relation to time is completely abstract, obviously it is even faster than light. On the real human mind, however, this does not apply. Neither our perceptions nor our imaginations can be absolutely spatial, although *our mind* has a *tendency towards unification*—obviously because this was phylogenetically favorable for survival, since disagreement inhibits action. Even if a person (after an operation, which cuts the connecting fibers of the two cerebral hemispheres within the corpus callosum, so-called split brain) in special experimental situations can reportedly have two independent streams of consciousness [58], he does not notice this splitting, on the contrary, he feels himself a unity. Nevertheless, without spatiality the mind cannot get by in a spatial world. Not only our perceptions, also our imaginations have top and bottom, front and rear. In fact, we notice that our thinking takes time, but how the relation is between consciousness and time, remains a secret to us to a large extent. 

## 7. Mind and Brain Are Not Separate

The Platonic-Christian idea is that the activity of the mind precedes that of the brain, but since the mind is envisaged as immaterial—meaning that it is thought to be without energy—this is not possible, for mind is order, and putting something into order requires energy. It could perhaps—theoretically—at the most be simultaneous, in reality however, our consciousness *follows* slightly behind most brain activities, because it requires a great expenditure in cooperative activity of neurons. An excitation of the neurons of our retina (which is a part of our brain extended forward) takes—depending on dark- or light-adaptation—about 60 ms or more to excite our visual cortex, and for the further processing towards a conscious perception, it takes even more time. With auditory and tactile perception, the conduction from the receptors to the cortex is slightly faster, but the processing in the brain requires approximately the same time and even longer with complex problems. The simple motor reaction time to a visual stimulus lasts in adults about two tenths of a second, in children longer. For a technical computer this would be very slow, but the strength of our brain does not lie in speed but in simultaneous processing in many partial systems with the utilization of great background knowledge. 

Let us just consider speaking: While one syllable is uttered—for which a highly precise motor organization with specific programs as well as auditory and tactile regulation via feedback is necessary, without which the sharp transitions of the frequencies at the beginning and at the end of the phonemes would be incomprehensible—one has to prepare not only the next syllable of the word but also the whole sentence. Consciousness has to supervise this enormous expenditure of information processing and, if necessary, has to intervene with corrections. Because of “parallel processing”, *i.e*., carrying out many steps of information processing simultaneously, statements about “before” and “after” and “simultaneous” of events in the brain are difficult [59], particularly since there are delays because of conduction and multiple chemical reactions. 

It is an error common among philosophers today that the natural sciences are only responsible for the outer aspect, not the *inner aspect*, that of perception, feeling, thinking and acting of man. We need these terms from inner experience in order to interpret our brain physiological measurements. On the other hand, at times we need physical methods, measuring things from outside, in order to help each other or at least understand each other. One can e.g., talk to somebody for quite some time about a Picasso painting from his pink period and gain the impression that the person one is talking with experiences something different, until one discovers by an objective test that he has some kind of color blindness (which he might not have been aware of). The opinion that we all have a similar consciousness is only partially correct. There are people who have something like a hole in the place where in others sympathy is located; this cannot easily be recognized but in the long run their behavior reveals it. Especially the combination of objective neurophysiology and subjective psychophysics delivers elucidating contributions to the understanding of the human mind, on both the perceptive side [60] and on the side of will, which we have given examples for in the book “*The Will and Its Brain*” [3]. Some philosophers, however, have understood that one has to know something about the real world, of its reception by the senses and its processing by the brain, in order to understand the mind better; they now speak of neurophilosophy; their discussions about how “*Gedankenexperimente*” (experiments in our mind’s eye) and other things work, can be found presented in an entertaining way with Dennett 2005 [9]. Quantum physics has shown that in the microphysical sphere, determination as *Newton* conceived it does not exist. There is, as Heisenberg [61] found, pure chance and unpredictability, and research has shown that chance can have effects even when one goes up into macrophysics, e.g., with the weather. The first natural scientists already acknowledged chance among the laws of nature. “Time is a child that plays”, wrote Heraclitus [62], and Democritus [63] went on to explain how, by random processes, order can develop, e.g., the assortment of pebbles on the beach. 

## 8. Decision Making

Decisions in the brain are mostly not made abruptly—except we are forced by the situation, for instance when skiing—rather they are made gradually. Even simple decisions—for instance the pressing of a right or a left button—need some time and a few seconds go by until we start the movement. The matter is different, if we are under time pressure or with rapt attention to a stimulus. This can be so because we were not yet absolutely determined or because the way from the frontal cortex does not go directly to motor cortex, which was earlier belief, but needs the cooperation of a phylogenetically old subcortical part of the brain that provides learned and stored movement programs, the *basal ganglia*. Functional magnetic resonance imaging then shows vague activities, which can be interpreted as if up to 10 s prior to the movement in the prefrontal brain a change in activity occurred that might lead to a decision a bit later, *i.e*., it looks as if an unconscious decision had been made [64]. This is not a sign of a lack of freedom, rather it signals insufficient attention or shortage of memory in conjunction with the absence of haste. The resulting movement is consciously controlled in any case. 

Some so-called modern neuroscientists are of the opinion that the principle of responsibility of man is untenable, for in the brain there is no leadership: “There is, in the brain, no location, where decisions are made.” This is an odd argument for, already in the visual cortex, the information is conducted from level to level and even on parallel pathways simultaneously to different higher stations of processing in order to finally converge and travel to the frontal cortex, where the information, along with signals from other senses and the “system of the needs” flows into the formation of will and thus for the leadership of the whole human being. Naturally, the frontal brain needs the cooperation of other parts of the brain; but even if the sites vary in which precedenced and subordinate decisions are made: Decisions exist in the brain. This is witnessed not only by our consciousness but also by our behavior. Scientists are obviously using the argument that in the brain, as far as we know, no “grandmother cells” exist, thus there are no neurons that are specialized on the recognition of our grandmother, rather the neurons in varying groupings can serve different purposes. This argument, however, could be used just as well for the impossibility of perception as against will. The fact that there is perception has never been denied by Neuroscientists. Not even Berkeley has claimed total agnosticism. Science, in any case is not compatible with total agnosticism. 

## 9. Some Thoughts on Freedom

Both principles—the hierarchical and the distributed/associative—are obviously realized in higher brains [65]. In its activity the brain can achieve astounding performances, which we are mostly not consciously aware of: For instance the visual perception of a figure on a moving background requires numerous multiplications carried out in a decentralized manner in the distributed system of the brain. On the other hand, our ability to mentally travel back the way in time of our own acting and experiencing and also the fates of companions in life, *i.e*., episodic memory, requires self-leadership with the high art of management, and this leadership is organized by the prefrontal cortex within the hierarchical system [66]—of course with the support of the distributed, associative system. The distributed system, the so-called modern neuroscientists are arguing with, is nothing new, it is discussed by brain researchers and cyberneticists for decades. But with its existence, which gives the brain great performance and high flexibility, a lack of freedom cannot be argued, on the contrary it is, similar as the hierarchical system, a rich source of freedom. We need both systems—If one asks oneself which cortical field, on the basis of its anatomical and neurophysiological data, could be brought into consideration as a candidate for a leadership function, one could also think of the parietal lobe, for in parietal cortex many sensory messages (visual, somatosensory and auditory) from the outer world come together [67] and serve for the regulation of attention. But this cortical region lacks the connections with the old system of the internal needs and drives with its messages from the inner world, limbic system and hypothalamus. It is true that with lesions of the parietal cortex we get disorders of attention but no loss of strategic planning. Combining both, the messages from the outer world and from the inner world takes place in the frontal lobe only. Therefore, it is not a matter of chance that the further evolution towards a higher planning and decision center has taken place in the prefrontal cortex—with responsibility, with conscience. But instead of acknowledging these facts that are in agreement with the ethical wisdom of mankind since the *Achsenzeit* (Jaspers’s axis time), since Heraclitus [62] and Kung-Tse [68] (Confucius), the spokespersons of the new irresponsibility try to argue away the autonomy of the mature man and are collecting, instead of arguments, allies for propaganda. 

Furthermore, the so-called modern neuroscientists think that the mere fact that the human mind has a physical basis proves that man is not free. This opinion is based on a term of freedom remote from nature: total freedom from nature. Such a concept is not realistic. Real freedom is relative, contingent, specific, gradual and naturally acquired. This freedom is not an illusion, assessment by others and self-experience fit together, e.g., in the case of being tired. Only with toxic disinhibition or lesion-related lack of self-criticism are there illusions of freedom (to the extent—e.g., with LSD-intoxication—of the illusion of being able to fly, with lethal consequences at times). In the normal range, however, our conscious estimation of freedom is usually realistic—which is to be expected according to the selection process of phylogeny. Skeptics of will should think about the fact that freedom of will in the sense of conscious, intended self-control of human behavior has experimentally been confirmed beyond any reasonable doubt (e.g., [69]). 

The organ of will lies in the frontal brain, more precisely in the prefrontal cortex, which, however, for its leadership needs messages from the rest of the brain about the outer world and the inner world, about the needs, and from memory, *etc*., and it delegates tasks to other parts of the brain. There is a division of tasks in the frontal cortex, as Karl Kleist discovered by examining brain injured patients from World War I, published in 1934 [70]: Mental drive and productive thinking are represented in the dorsolateral and the polar prefrontal cortex, conscience and emotional control in the orbital cortex. The leadership on our thinking is assisted by the frontal working memory, which was discovered by Jacobsen [71]. The frontomedial supplementary motor area (SMA), in which the Bereitschaftspotential prior to willful movements occurs, controls for the right moment to start a volitional movement. The SMA works closely together with the basal ganglia, which assist the cortex (among others with motor tasks and with speaking) with the help of programs learnt earlier. 

## 10. Conclusions

Consciousness and the unconscious in the brain always cooperate. Information processing in the brain needs space, time and energy; it is based on collaboration in a distributed system but striving for unity under the leadership of ethical will. 

I would like to end here, repeating that the unconscious is not the “bad something” Freud had conceived, a something that plays tricks on us. It is rather the groundworker for consciousness, the staff that supports the leader. And the leader is: The conscious, reasoned free will. One clinical experience has still to be mentioned. Above in Section 6 on awareness, we mentioned that a split brain patient does not feel “split” but he feels himself a unity. It surely is oversimplified, when we are asking whether consciousness is in the left or in the right hemisphere. And the answer is, with alphanumeric stuff it is in the left hemisphere, with spatial constructive agendas it is in the right hemisphere. But one thing is clear: Consciousness needs a hemisphere. We know from epileptic patients that a focal seizure can occur, and the patient is still conscious. The seizure activity can occupy the whole sensorimotor cortex, we call this a “hemi grand mal”, and the patient is still conscious. However as soon as the seizure generalizes—and generalizing means that the seizure activity travels through the corpus callosum to the other hemisphere—the patient loses consciousness.
